# Microbial transformation of widely used pharmaceutical and personal care product compounds

**DOI:** 10.12688/f1000research.21827.1

**Published:** 2020-02-21

**Authors:** Abigail W. Porter, Sarah J. Wolfson, Max Häggblom, Lily Y. Young

**Affiliations:** 1 Department of Environmental Sciences, School of Environmental and Biological Sciences, Rutgers, the State University of New Jersey, New Brunswick, NJ, USA; 2 Department of Systems and Computational Biology, Albert Einstein College of Medicine, Bronx, NY, USA; 3 Department of Biochemistry and Microbiology, School of Environmental and Biological Sciences, Rutgers, the State University of New Jersey, New Brunswick, NJ, USA

**Keywords:** Biotransformation, anaerobic O-demethylation, anaerobic O-methylation, pharmaceutical biodegradation

## Abstract

Pharmaceutical and personal care products (PPCPs) are commonly used chemicals that are increasingly detected in urban-impacted environments, particularly those receiving treated wastewater. PPCPs may have toxicological effects on the macrofauna that are exposed through contaminated water; thus, there is interest in microbially mediated transformations that may degrade PPCPs. This review discusses specific examples of PPCP transformations that may occur in anoxic environments, including O-methylation and O-demethylation.

## Introduction

Pharmaceutical and personal care products (PPCPs) contain chemicals that are widely distributed in surface waters, sediment, and soil ^1, 2^. Pharmaceuticals enter wastewater treatment plants through ingestion and subsequent excretion ^3^, through improper disposal down a household drain ^4^, or from pharmaceutical manufacturing plant discharge ^5^. Wastewater treatment plants are not designed to remove these complex organic contaminants, which can result in incomplete PPCP removal. A major concern is, therefore, that treated effluent may contain low concentrations of PPCPs that can enter receiving waters or soils when biosolids are used as fertilizer ^6– 8^.

A range of adverse effects has been reported for wildlife that is exposed to treated effluent. When released into the environment, pharmaceuticals can be toxic ^9^ or can cause unwanted physiological responses to non-target organisms, including endocrine disruption (e.g. feminization of fish), altered development of aquatic organisms including fish and frogs, and changes to behavior ^10– 13^. In addition, bioaccumulation in aquatic organisms is a concern, particularly in fish intended for human consumption ^14^. Not only are these findings a potential public health problem, but they also raise concerns about the health of the ecosystem and overall water quality. While the concentrations of an individual chemical may be in the ng L ^-1^ range (for example, see 15), PPCPs are typically found in wastewater as complex mixtures and may have additive effects that remain to be understood.

## Microbial toxicity

Some PPCPs are designed specifically to have antagonist effects against microorganisms. Notably, this includes antibiotics, preservatives (e.g. parabens), and antimicrobial compounds (e.g. triclosan and triclocarban). Others may have unexpected inhibitory effects. Ibuprofen, for example, has been shown to inhibit the growth of a variety of microorganisms ^16^. Pharmaceuticals such as propranolol, diphenhydramine, and diclofenac sodium have also been reported to have inhibitory effects on the methanogenic microbial community found in anaerobic digesters ^17, 18^. Furthermore, the metabolites produced during microbial transformation of pharmaceuticals are not always further degraded ^18– 20^ and could also have negative effects on the microbial community. Alternatively, the microbial community may still carry out the desired function, such as methanogenesis, but the microbial community composition may be altered or enriched for antibiotic resistance genes ^21, 22^. In addition, these metabolites can still be pharmacologically active and can exhibit toxicity to eukaryotic organisms, although these effects have not yet been documented in prokaryotic organisms such as bacteria and archaea ^23, 24^. As prokaryotes provide ecosystem services for all environments, the effects of PPCPs and their metabolites on prokaryotes are valuable to know.

## Biodegradation

PPCPs enter changing environmental conditions and encounter diverse microbial communities as they pass from households through the wastewater treatment process and ultimately into the environment. The initial stages of wastewater treatment are designed to first use well-oxygenated units to support aerobic degradation. Later in the process, further degradation of the sludge solids takes place in anaerobic digester units that promote a fermenting and methanogenic community operating under low oxidation-reduction potential (<–350 mV). Treated wastewater effluent is released into oxic surface water; however, some PPCPs will eventually migrate into anoxic sediments ^25– 28^. In freshwater systems, nitrate, iron, or carbonate are predominant electron acceptors available for respiration, whereas coastal marine waters would additionally have sulfate as a respiratory electron acceptor. These different conditions, therefore, support diverse microbial communities that may also be capable of divergent biochemical mechanisms for biodegradation in surface waters and anoxic sediments. This must all be taken into account when modeling the environmental fate of PPCPs, as degradation might be more likely to occur, proceed to a greater extent, or produce different intermediates depending on the location. Naproxen transformation, for example, has been shown to occur under sulfate-reducing and methanogenic conditions in constructed wetlands, estuarine sediment, and anaerobic digester sludge, yet nitrate-reducing conditions in constructed wetlands yielded little transformation activity ^20, 29^. Oxybenzone, in contrast, was transformed under aerobic, nitrate-reducing, iron-reducing, sulfate-reducing, and methanogenic conditions ^30, 31^. PPCPs have diverse chemical structures that underscore the need for a broader understanding of how microbes in different environments will metabolize the different classes of compounds. This is valuable for predicting potential activity in the environment, as partial microbial transformations may make the original PPCP undetectable by standard methods, yet the new transformation product may have ecotoxicological effects.

## Fate in wastewater treatment plants and receiving aquatic habitats

There have been many reports of PPCP removal during the biological stages of wastewater treatment (for reviews, see 32, 33) or quantifying PPCPs in effluent-impacted water and sediment ^2, 34^. Treated wastewater effluents are one of the main pathways by which PPCPs enter watersheds. While some removal during wastewater treatment can be attributed to the biological activity of microorganisms, there are few simplified consortia or pure cultures available to demonstrate the biochemistry involved in PPCP transformation. Without biochemical evidence, it is difficult to determine if the PPCP in question has been mineralized ^35^, lost due to abiotic processes such as sorption to solids ^36– 38^, or transformed into unknown metabolites ^31, 39^.

Some anaerobic transformation reactions may lead to persistent metabolites that present additional environmental problems. Nonylphenol and octylphenol, for example, are produced from the sequential removal of ethoxyl groups from the nonionic surfactants nonylphenol polyethoxylate and octylphenol polyethoxylate, as shown in Table 1 ^40, 41^. These metabolites have been shown to mimic estrogen ^13^ and are frequently detected in wastewater treatment systems and in the aquatic environment ^2^. The genes and biochemical intermediates for nonylphenol and octylphenol degradation have been reported under aerobic conditions ^42– 44^; however, only limited data exist regarding their fate under anaerobic conditions and the biochemical pathways are largely unknown ^46– 49^. With the identification of nonylphenol and octylphenol as persistent metabolites with toxicological effects, it is now imperative to monitor their concentrations in the environment and quantify their potential estrogenic impact.

**Table 1.** Anaerobic and aerobic transformation reactions may lead to persistent metabolites. Specific examples of pharmaceutical and personal care products with corresponding transformation products are shown.

Parent compound	Transformation product	References
**O-Demethylation**		
**Naproxen** Over-the-counter non-steroidal anti- inflammatory drug 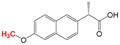	6-O-Desmethylnaproxen 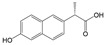	20, 31
**Guaifenesin** Expectorate 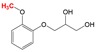	3-(2-hydroxyphenoxy) propane-1,2-diol 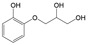	31
**Oxybenzone** UV light absorber Found in sunscreens and plastics 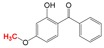	2,4-Dihydroxybenzophenone 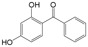	31
**Methylparaben** Preservative in cosmetics, pharmaceuticals, and food 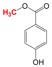	4-Hydroxybenzoic acid 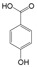	31
**N-Demethylation**		
**Diphenhydramine** Anti-histamine 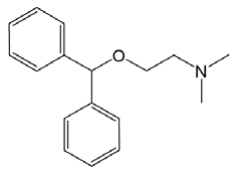	N-Desmethyl diphenhydramine 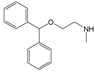	18
**O-Methylation**		
**Bisphenol A (BPA)** Plastic precursor 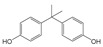	BPA monomethyl ether (left), BPA dimethyl ether (right) 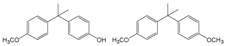	45
**De-ethoxylation**		
**Octylphenol and** **nonylphenol** **polyethoxylate** Nonionic surfactant 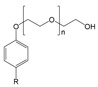	Octylphenol or nonylphenol 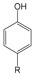	40

Other types of anaerobic biotransformation reactions include O-demethylation. Recently, we reported on the complex microbial strategy of naproxen transformation by a methanogenic consortium enriched from anaerobic digester sludge ^20^. The methanogenic consortium O-demethylated naproxen to form the persistent metabolite 6-O-desmethylnaproxen, which is illustrated in Table 1. Acetogenic bacteria were responsible for this step and produced acetate that subsequently enriched for a population of syntrophic acetate-oxidizing bacteria. The latter supported a methanogenic community that produced the amount of methane that was consistent with O-demethylation ^20^. This model is an example of an anaerobic microbial food web that was supported through pharmaceutical biotransformation.

Similarly, diphenhydramine can be transformed by anaerobic digester sludge microbes via N-demethylation to N-desmethyl diphenhydramine (see Table 1; ^18^), a metabolite formerly known to be generated only by mammals and fungi ^50^. While the parent compound, diphenhydramine, suppressed both fermentative and methanogenic activity in the anaerobic digester community, the metabolite suppressed only methanogenic activity. In contrast, there was negligible toxicity of naproxen and 6-O-desmethylnaproxen to the same community ^20^. These differences highlight how chemically different PPCPs and their transformation products may have different effects on the same microbial community, further underscoring the complexity of the fate and effect of the PPCPs.

While anaerobic O-demethylation of aromatic compounds has been well established (see 51), less is known about this transformation in PPCPs. We have evidence that PPCPs with diverse uses and chemical structures but share a phenylmethyl ether functional group can be transformed via O-demethylation ^31^. Microbial communities enriched under both methanogenic and sulfate-rich conditions showed this capability when provided with naproxen, guaifenesin, methylparaben, or oxybenzone ^31^. The sulfate-rich cultures formed O-demethylated metabolites, shown in Table 1, that were not further degraded. A similar pattern was observed in the methanogenic cultures ^31^.

In contrast, many phenolic compounds can be transformed by microbial O-methylation (see 52). For example, bacteria are able to O-methylate bisphenol A (BPA) to its monomethyl and dimethyl ether derivatives, as pictured in Table 1 (BPA MME and BPA DME, respectively) ^45^, resulting in metabolites with increased toxicity as shown from differences in survival and occurrence of developmental lesions in developing zebrafish embryos exposed to BPA, BPA MME, and BPA DME. The monomethyl and dimethyl ether derivatives were more toxic than BPA, resulting in increased mortality. Furthermore, exposure to either of the O-methylated metabolites resulted in an increase in the incidence of developmental lesions as compared to BPA exposure ^45^. These data illustrate a new mechanism for the microbial transformation of BPA, producing metabolites warranting further study to understand their prevalence and effects in the environment. In addition, the O-methylated transformation products could serve as potential substrates for O-demethylation by the organisms described above ^31, 51^. The interconversion between O-methylated and O-demethylated forms thus presents a mechanism by which a PPCP compound can be transformed in one environment and the original parent compound regenerated by microbes that are active in another environment. This is similar to reports of flame-retardant and antimicrobial compound transformations that have been described in plants ^53, 54^.

## Predicting anaerobic biodegradation

Identifying common functional groups may serve as a basis for predicting transformation products. Gulde *et al*. ^55^ used a systematic approach to identify potential metabolites of PPCPs that contain an amine group, applying this method to predicting reactions in aerobic activated sludge. Alternatively, we used a culture-based approach to examine the range of O-demethylation substrates in anoxic sediments and anaerobic wastewater digestion ^31^. Gonzalez-Gil *et al*. ^56^ used enzyme assays to examine co-metabolic transformations of diverse PPCPs, including naproxen, nonylphenol, octylphenol, triclosan, and BPA, that were mediated by acetate kinase. The extent to which transformation occurred varied with the substrate from 10–90% and suggested the involvement of additional transformation pathways ^56^, which could lead to a mixture of different transformation products existing from the same parent compound. Laboratory-based assays, such as those conducted by Gonzalez-Gil *et al*. ^56^ and Wolfson *et al*. ^31^, represent a starting point for the identification of potential metabolites, although it may not be representative of the dominant transformation mechanism that occurs in the environment. Likewise, the microbial community composition may have an effect on PPCP transformations, especially under methanogenic conditions ^20, 57^. Additionally, the effects that mixtures of PPCPs and associated transformation products will have on microbial community function cannot be overlooked.

## Future directions for PPCP removal

Recognition of the expanding extent of PPCP contamination has stimulated the search for solutions that remove pharmaceuticals from wastewater before they can reach the environment, including technologies like advanced oxidation processes and membrane bioreactors ^58– 62^. These new technologies have shown promise with higher removal rates in pilot treatment plants than with conventional treatment ^61, 63^. In combination with increased removal efficiencies, re-designing PPCPs to promote biodegradability could lead to a reduction in the environmental load in the future ^64, 65^. Understanding transformation products is important not only to the health of impacted aquatic ecosystems and humans but also for monitoring the safety of reclaimed wastewater reuse (for review, see 66) and for the accuracy of wastewater-based epidemiology to follow human health and pharmaceutical use ^67– 70^.

## Conclusions

An Organization for Economic Cooperation and Development report projects that sales of chemicals worldwide will increase by 3% annually between now and 2050 ^71^, thus providing a steady stream of diverse chemical structures that may be entering wastewater treatment and the environment. Given that pharmaceuticals are used daily throughout the world, their release into the environment is both a public and an environmental health concern. Understanding not only the microbial transformation processes but also the metabolites that are formed is essential for comprehensive accounting of pharmaceuticals and potential pharmaceutically active compounds in the environment. The environmental context, be it the engineered anaerobic digester or freshwater or estuarine sediments that are impacted by treated wastewater, is critical for understanding potential microbial activities and biodegradation mechanisms to determine if biodegradation will occur or if potential metabolites may form and accumulate under the given redox conditions. This knowledge may provide solutions to remove these pharmaceuticals during wastewater treatment and prevent environmental deposition as well as to understand environmental processes that may occur to remove pharmaceuticals that have already entered the environment.

## References

[1] Cantwell MG Katz DR Sullivan JC: Spatial patterns of pharmaceuticals and wastewater tracers in the Hudson River Estuary. *Water Res.* 2018;137:335–43. 10.1016/j.watres.2017.12.044 29571111 PMC6582947

[2] Kolpin DW Furlong ET Meyer MT: Pharmaceuticals, hormones, and other organic wastewater contaminants in U.S. streams, 1999-2000: a national reconnaissance. *Environ Sci Technol.* 2002;36(6):1202–11. 10.1021/es011055j 11944670

[3] Xia K Bhandari A Das K: Occurrence and fate of pharmaceuticals and personal care products (PPCPs) in biosolids. *J Environ Qual.* 2005;34(1):91–104. 10.2134/jeq2005.0091 15647538

[4] Jelic A Rodriguez-Mozaz S Barceló D: Impact of in-sewer transformation on 43 pharmaceuticals in a pressurized sewer under anaerobic conditions. *Water Res.* 2015;68:98–108. 10.1016/j.watres.2014.09.033 25462720

[5] Fick J Söderström H Lindberg RH: Contamination of surface, ground, and drinking water from pharmaceutical production. *Environ Toxicol Chem.* 2009;28(12):2522–7. 10.1897/09-073.1 19449981

[6] Biel-Maeso M Corada-Fernández C Lara-Martín PA: Removal of personal care products (PCPs) in wastewater and sludge treatment and their occurrence in receiving soils. *Water Res.* 2019;150:129–39. 10.1016/j.watres.2018.11.045 30508710

[7] Kinney CA Furlong ET Zaugg SD: Survey of organic wastewater contaminants in biosolids destined for land application. *Environ Sci Technol.* 2006;40(23):7207–15. 10.1021/es0603406 17180968

[8] Sabourin L Beck A Duenk PW: Runoff of pharmaceuticals and personal care products following application of dewatered municipal biosolids to an agricultural field. *Sci Total Environ.* 2009;407(16):4596–604. 10.1016/j.scitotenv.2009.04.027 19464726

[9] Watanabe H Tamura I Abe R: Chronic toxicity of an environmentally relevant mixture of pharmaceuticals to three aquatic organisms (alga, daphnid, and fish). *Environ Toxicol Chem.* 2016;35(4):996–1006. 10.1002/etc.3285 26472177

[10] Bringolf RB Heltsley RM Newton TJ: Environmental occurrence and reproductive effects of the pharmaceutical fluoxetine in native freshwater mussels. *Environ Toxicol Chem.* 2010;29(6):1311–8. 10.1002/etc.157 20821574

[11] Dzieweczynski TL Campbell BA Kane JL: Dose-dependent fluoxetine effects on boldness in male Siamese fighting fish. *J Exp Biol.* 2016;219(Pt 6):797–804. 10.1242/jeb.132761 26985051

[12] Weinberger J 2nd Klaper R: Environmental concentrations of the selective serotonin reuptake inhibitor fluoxetine impact specific behaviors involved in reproduction, feeding and predator avoidance in the fish *Pimephales promelas* (fathead minnow). *Aquat Toxicol.* 2014;151:77–83. 10.1016/j.aquatox.2013.10.012 24210950 PMC3989372

[13] White R Jobling S Hoare SA: Environmentally persistent alkylphenolic compounds are estrogenic. *Endocrinology.* 1994;135(1):175–82. 10.1210/endo.135.1.8013351 8013351

[14] Huerta B Rodriguez-Mozaz S Lazorchak J: Presence of pharmaceuticals in fish collected from urban rivers in the U.S. EPA 2008-2009 National Rivers and Streams Assessment. *Sci Total Environ.* 2018;634:542–9. 10.1016/j.scitotenv.2018.03.387 29635196 PMC6097189

[15] Archer E Petrie B Kasprzyk-Hordern B: The fate of pharmaceuticals and personal care products (PPCPs), endocrine disrupting contaminants (EDCs), metabolites and illicit drugs in a WWTW and environmental waters. *Chemosphere.* 2017;174:437–46. 10.1016/j.chemosphere.2017.01.101 28187390

[16] Obad J Šušković J Kos B: Antimicrobial activity of ibuprofen: new perspectives on an "Old" non-antibiotic drug. *Eur J Pharm Sci.* 2015;71:93–8. 10.1016/j.ejps.2015.02.011 25708941

[17] Fountoulakis MS Stamatelatou K Lyberatos G: The effect of pharmaceuticals on the kinetics of methanogenesis and acetogenesis. *Bioresour Technol.* 2008;99(15):7083–90. 10.1016/j.biortech.2008.01.008 18280143

[18] Wolfson SJ Porter AW Villani TS: The antihistamine diphenhydramine is demethylated by anaerobic wastewater microorganisms. *Chemosphere.* 2018;202:460–6. 10.1016/j.chemosphere.2018.03.093 29579680

[19] Helbling DE Hollender J Kohler HP: High-throughput identification of microbial transformation products of organic micropollutants. *Environ Sci Technol.* 2010;44(17):6621–7. 10.1021/es100970m 20799730

[20] Wolfson SJ Porter AW Campbell JK: Naproxen Is Transformed Via Acetogenesis and Syntrophic Acetate Oxidation by a Methanogenic Wastewater Consortium. *Microb Ecol.* 2018;76(2):362–71. 10.1007/s00248-017-1136-2 29327072

[21] Carey DE Zitomer DH Hristova KR: Triclocarban Influences Antibiotic Resistance and Alters Anaerobic Digester Microbial Community Structure. *Environ Sci Technol.* 2016;50(1):126–34. 10.1021/acs.est.5b03080 26588246

[22] Fujimoto M Carey DE McNamara PJ: Metagenomics reveal triclosan-induced changes in the antibiotic resistome of anaerobic digesters. *Environ Pollut.* 2018;241:1182–90. 10.1016/j.envpol.2018.06.048 30029328

[23] Celiz MD Tso J Aga DS: Pharmaceutical metabolites in the environment: analytical challenges and ecological risks. *Environ Toxicol Chem.* 2009;28(12):2473–84. 10.1897/09-173.1 19663539

[24] López-Serna R Petrović M Barceló D: Occurrence and distribution of multi-class pharmaceuticals and their active metabolites and transformation products in the Ebro River basin (NE Spain). *Sci Total Environ.* 2012;440:280–9. 10.1016/j.scitotenv.2012.06.027 22809787

[25] Cantwell MG Wilson BA Zhu J: Temporal trends of triclosan contamination in dated sediment cores from four urbanized estuaries: evidence of preservation and accumulation. *Chemosphere.* 2010;78(4):347–52. 10.1016/j.chemosphere.2009.11.021 20006371

[26] Hajj-Mohamad M Aboulfadl K Darwano H: Wastewater micropollutants as tracers of sewage contamination: analysis of combined sewer overflow and stream sediments. *Environ Sci Process Impacts.* 2014;16(10):2442–50. 10.1039/c4em00314d 25189851

[27] Lara-Martín PA Renfro AA Cochran JK: Geochronologies of pharmaceuticals in a sewage-impacted estuarine urban setting (Jamaica Bay, New York). *Environ Sci Technol.* 2015;49(10):5948–55. 10.1021/es506009v 25884477

[28] Li X Doherty AC Brownawell B: Distribution and diagenetic fate of synthetic surfactants and their metabolites in sewage-impacted estuarine sediments. *Environ Pollut.* 2018;242(Pt A):209–18. 10.1016/j.envpol.2018.06.064 29980039

[29] He Y Sutton NB Rijnaarts HHM: Pharmaceutical biodegradation under three anaerobic redox conditions evaluated by chemical and toxicological analyses. *Sci Total Environ.* 2018;618:658–64. 10.1016/j.scitotenv.2017.07.219 29055590

[30] Liu YS Ying GG Shareef A: Biodegradation of the ultraviolet filter benzophenone-3 under different redox conditions. *Environ Toxicol Chem.* 2012;31(2):289–95. 10.1002/etc.749 22095591

[31] Wolfson SJ Porter AW Villani TS: Pharmaceuticals and Personal Care Products Can Be Transformed by Anaerobic Microbiomes in the Environment and in Waste-Treatment Processes. *Environ Toxicol Chem.* 2019;38(7):1585–93. 10.1002/etc.4406 30883883

[32] Alvarino T Lema J Omil F: Trends in organic micropollutants removal in secondary treatment of sewage. *Rev Environ Sci Biotechnol.* 2018;17:447–69. 10.1007/s11157-018-9472-3

[33] Tiwari B Sellamuthu B Ouarda Y: Review on fate and mechanism of removal of pharmaceutical pollutants from wastewater using biological approach. *Bioresour Technol.* 2017;224:1–12. 10.1016/j.biortech.2016.11.042 27889353

[34] Bradley PM Journey CA Romanok KM: Expanded Target-Chemical Analysis Reveals Extensive Mixed-Organic-Contaminant Exposure in U.S. Streams. *Environ Sci Technol.* 2017;51(9):4792–802. 10.1021/acs.est.7b00012 28401767 PMC5695041

[35] Wu Y Sun Q Wang YW: Comparative studies of aerobic and anaerobic biodegradation of methylparaben and propylparaben in activated sludge. *Ecotoxicol Environ Saf.* 2017;138:25–31. 10.1016/j.ecoenv.2016.12.017 27992847

[36] Blair B Nikolaus A Hedman C: Evaluating the degradation, sorption, and negative mass balances of pharmaceuticals and personal care products during wastewater treatment. *Chemosphere.* 2015;134:395–401. 10.1016/j.chemosphere.2015.04.078 25985097

[37] Cantwell MG Katz DR Sullivan JC: Selected pharmaceuticals entering an estuary: Concentrations, temporal trends, partitioning, and fluxes. *Environ Toxicol Chem.* 2016;35(11):2665–73. 10.1002/etc.3452 27062058

[38] Martín J Santos JL Aparicio I: Pharmaceutically active compounds in sludge stabilization treatments: anaerobic and aerobic digestion, wastewater stabilization ponds and composting. *Sci Total Environ.* 2015;503-504:97–104. 10.1016/j.scitotenv.2014.05.089 24909712

[39] Gasser G Pankratov I Elhanany S: Field and laboratory studies of the fate and enantiomeric enrichment of venlafaxine and O-desmethylvenlafaxine under aerobic and anaerobic conditions. *Chemosphere.* 2012;88(1):98–105. 10.1016/j.chemosphere.2012.02.074 22445391

[40] Giger W Brunner PH Schaffner C: 4-Nonylphenol in sewage sludge: accumulation of toxic metabolites from nonionic surfactants. *Science.* 1984;225(4662):623–5. 10.1126/science.6740328 6740328

[41] Lu J Jin Q He Y: Biodegradation of nonylphenol polyethoxylates by denitrifying activated sludge. *Water Res.* 2008;42(4–5):1075–82. 10.1016/j.watres.2007.09.031 17980399

[42] Kolvenbach BA Corvini PF: The degradation of alkylphenols by *Sphingomonas* sp. strain TTNP3 - a review on seven years of research *N Biotechnol.* 2012;30(1):88–95. 10.1016/j.nbt.2012.07.008 22842087

[43] Porter AW Hay AG: Identification of *opdA*, a gene involved in biodegradation of the endocrine disrupter octylphenol. *Appl Environ Microbiol.* 2007;73(22):7373–9. 10.1128/AEM.01478-07 17890335 PMC2168194

[44] Wang Z Yang Y He T: Change of microbial community structure and functional gene abundance in nonylphenol-degrading sediment. *Appl Microbiol Biotechnol.* 2015;99(7):3259–68. 10.1007/s00253-014-6222-5 25421563

[45] McCormick JM Van Es T Cooper KR: Microbially mediated O-methylation of bisphenol A results in metabolites with increased toxicity to the developing zebrafish (Danio rerio) embryo. *Environ Sci Technol.* 2011;45(15):6567–74. 10.1021/es200588w 21678910

[46] Chang BV Lu ZJ Yuan SY: Anaerobic degradation of nonylphenol in subtropical mangrove sediments. *J Hazard Mater.* 2009;165(1–3):162–7. 10.1016/j.jhazmat.2008.09.085 18990492

[47] Chang BV Chiang F Yuan SY: Anaerobic degradation of nonylphenol in sludge. *Chemosphere.* 2005;59(10):1415–20. 10.1016/j.chemosphere.2004.12.055 15876384

[48] de Weert J Viñas M Grotenhuis T: Aerobic nonylphenol degradation and nitro-nonylphenol formation by microbial cultures from sediments. *Appl Microbiol Biotechnol.* 2010;86(2):761–71. 10.1007/s00253-009-2394-9 20043151 PMC2825322

[49] Wang Z Yang Y Dai Y: Anaerobic biodegradation of nonylphenol in river sediment under nitrate- or sulfate-reducing conditions and associated bacterial community. *J Hazard Mater.* 2015;286:306–14. 10.1016/j.jhazmat.2014.12.057 25590825

[50] Moody JD Heinze TM Hansen EB Jr: Metabolism of the ethanolamine-type antihistamine diphenhydramine (Benadryl) by the fungus *Cunninghamella elegans*. *Appl Microbiol Biotechnol.* 2000;53(3):310–5. 10.1007/s002530050026 10772471

[51] Frazer AC: O-Demethylation and Other Transformations of Aromatic Compounds by Acetogenic Bacteria.In: Drake, H.L. (Ed.), Acetogenesis. Springer US,1994;445–483. 10.1007/978-1-4615-1777-1_17

[52] Haggblom MM: Microbial breakdown of halogenated aromatic pesticides and related compounds. *FEMS Microbiol Lett.* 1992;9(1):29–71. 10.1111/j.1574-6968.1992.tb05823.x 1389314

[53] Fu Q Liao C Du X: Back Conversion from Product to Parent: Methyl Triclosan to Triclosan in Plants. *Environ Sci Technol Lett.* 2018;5:181–5. 10.1021/acs.estlett.8b00071

[54] Hou X Yu M Liu A: Biotransformation of tetrabromobisphenol A dimethyl ether back to tetrabromobisphenol A in whole pumpkin plants. *Environ Pollut.* 2018;241:331–8. 10.1016/j.envpol.2018.05.075 29843015 PMC6351071

[55] Gulde R Meier U Schymanski EL: Systematic Exploration of Biotransformation Reactions of Amine-Containing Micropollutants in Activated Sludge. *Environ Sci Technol.* 2016;50(6):2908–20. 10.1021/acs.est.5b05186 26864277

[56] Gonzalez-Gil L Carballa M Lema JM: Cometabolic Enzymatic Transformation of Organic Micropollutants under Methanogenic Conditions. *Environ Sci Technol.* 2017;51(5):2963–71. 10.1021/acs.est.6b05549 28198617

[57] Cetecioglu Z Ince B Orhon D: Anaerobic sulfamethoxazole degradation is driven by homoacetogenesis coupled with hydrogenotrophic methanogenesis. *Water Res.* 2016;90:79–89. 10.1016/j.watres.2015.12.013 26724442

[58] Kanakaraju D Glass BD Oelgemöller M: Advanced oxidation process-mediated removal of pharmaceuticals from water: A review. *J Environ Manage.* 2018;219:189–207. 10.1016/j.jenvman.2018.04.103 29747102

[59] Ma J Dai R Chen M: Applications of membrane bioreactors for water reclamation: Micropollutant removal, mechanisms and perspectives. *Bioresour Technol.* 2018;269:532–43. 10.1016/j.biortech.2018.08.121 30195697

[60] Prasse C Stalter D Schulte-Oehlmann U: Spoilt for choice: A critical review on the chemical and biological assessment of current wastewater treatment technologies. *Water Res.* 2015;87:237–70. 10.1016/j.watres.2015.09.023 26431616

[61] Radjenović J Petrović M Barceló D: Fate and distribution of pharmaceuticals in wastewater and sewage sludge of the conventional activated sludge (CAS) and advanced membrane bioreactor (MBR) treatment. *Water Res.* 2009;43(3):831–41. 10.1016/j.watres.2008.11.043 19091371

[62] Stasinakis AS: Use of Selected Advanced Oxidation Processes (AOPs) for Wastewater Treatment – a Mini Review. *Global NEST Journal.* 2008;10:376–85. Reference Source

[63] Angeles LF Mullen RA Huang IJ: Assessing pharmaceutical removal and reduction in toxicity provided by advanced wastewater treatment systems. *Environ Sci Water Res Technol.* 2020;6:62–77. 10.1039/C9EW00559E

[64] Kümmerer K Dionysiou DD Olsson O: Reducing aquatic micropollutants - Increasing the focus on input prevention and integrated emission management. *Sci Total Environ.* 2019;652:836–50. 10.1016/j.scitotenv.2018.10.219 30380490

[65] Rastogi T Leder C Kümmerer K: Re-Designing of Existing Pharmaceuticals for Environmental Biodegradability: A Tiered Approach with β-Blocker Propranolol as an Example. *Environ Sci Technol.* 2015;49(19):11756–63. 10.1021/acs.est.5b03051 26291878

[66] Christou A Agüera A Bayona JM: The potential implications of reclaimed wastewater reuse for irrigation on the agricultural environment: The knowns and unknowns of the fate of antibiotics and antibiotic resistant bacteria and resistance genes - A review. *Water Res.* 2017;123:448–67. 10.1016/j.watres.2017.07.004 28689129

[67] Baker DR Barron L Kasprzyk-Hordern B: Illicit and pharmaceutical drug consumption estimated via wastewater analysis. Part A: Chemical analysis and drug use estimates. *Sci Total Environ.* 2014;487:629–41. 10.1016/j.scitotenv.2013.11.107 24377678

[68] Choi PM Tscharke B Samanipour S: Social, demographic, and economic correlates of food and chemical consumption measured by wastewater-based epidemiology. *Proc Natl Acad Sci U S A.* 2019;116(43):21864–73. 10.1073/pnas.1910242116 31591193 PMC6815118

[69] Daughton CG: Monitoring wastewater for assessing community health: Sewage Chemical-Information Mining (SCIM). *Sci Total Environ.* 2018;619–620:748–64. 10.1016/j.scitotenv.2017.11.102 29161600 PMC6091531

[70] O'Brien JW Banks APW Novic AJ: Impact of in-Sewer Degradation of Pharmaceutical and Personal Care Products (PPCPs) Population Markers on a Population Model. *Environ Sci Technol.* 2017;51(7):3816–23. 10.1021/acs.est.6b02755 28244310

[71] OECD, OECD Environmental Outlook to 2050: The Consequences of Inaction. OECD Publishing, Paris. 2012 10.1787/9789264122246-en

